# Ripping the Myth: Patients’ Symptomatic Descriptions of Acute Thoracic Aortic Dissection

**DOI:** 10.51894/001c.6783

**Published:** 2018-04-27

**Authors:** Meghna Nagabhushan, James Webley

**Affiliations:** 1 Staff Emergency Physician, Medical City Alliance, Fort Worth, Texas; 2 Clinical Assistant Professor, McLaren Oakland Hospital Emergency Medicine and Genesys Regional Medical Center, Emergency Medicine Residency Program, Pontiac and Grand Blanc, MI

**Keywords:** emergency medicine, patient symptoms, thoracic aortic aneurism

## Abstract

**CONTEXT:**

The objective of this retrospective project was to assess the frequency with which patients presenting to an emergency department had used the descriptive terms “ripping” and “tearing” to describe their symptoms from later-confirmed acute thoracic aortic dissection.

**METHODS:**

The authors conducted a retrospective chart review from 58 patients who had presented to two suburban and urban emergency departments with suspected acute thoracic dissection between 1997 and 2015. They reviewed charts for patients’ pain descriptors in ambulance personnel records and initial notes and dictations from ED triage nurses, staff nurses, and physicians. These pieces of documentation would have been made before the diagnosis of acute thoracic aortic dissection could been confirmed.

**RESULTS:**

The authors identified a sample subset of 29 (50% of total charts pulled) patients later confirmed to have had an acute thoracic aorta dissection. They found that no sample patients used either the descriptors “ripping” or “tearing” when communicating their presenting symptoms. In this paper, the authors will provide several alternative terms patients have been shown to offer for this life-threatening condition.

**CONCLUSIONS:**

Although the terms “ripping” and “tearing” have historically been associated with acute thoracic aortic dissections, these project results indicate that clinicians may consider other descriptive symptomatic terms from patients when evaluating patients’ symptoms for this potential life-threatening condition.

## INTRODUCTION

Acute dissection of the thoracic aorta is a life-threatening problem for patients who have sustained a tear in the innermost intima lining of the aorta.^1^ The patient’s hydrodynamic pressure of blood flow then expands the tear proximally, distally, or in both directions into and through the other layers of the artery. As the rent (i.e., defect) in the aortic wall dissects its way along the aorta, this condition may compromise other connecting arteries (e.g., carotids, subclavian, renal, spinal arteries) depending on the direction and length of the tear.^1^

Additionally, a proximal tear into the root of the aorta may dissect into: a) the coronary arteries causing a myocardial infarction, b) damaged aortic valve causing sudden congestive heart failure, or c) break free into the pericardium causing abrupt catastrophic death from pericardial tamponade.^1^ The large number of organs that may be involved during this disease process causes patients’ associated symptoms to vary widely, frequently confusing clinicians.^2^

In 2000, a group of experts in The International Registry of Acute Aortic Dissection (IRAD) found in a sample with 464 TAD patients that severe pain (95.5%) was their most common symptom.^3^ The pain was, in fact, more commonly described as “worst-ever” (90.6%), “abrupt onset” (84.8%), and “sharp” (64.4%).^3^ In this larger study, “ripping” or “tearing” researchers identified these terms in 50.6% of sample cases.^3^

Acute thoracic aortic dissection (TAD) has claimed the lives of numerous famous individuals including John Ritter, Lucille Ball, Jonathon Larson (writer of *Rent*), and King George II of England. The condition continues to afflict 5,000-10,000 people a year in the US.^1^ TAD has a shocking mortality rate estimated at 40% initially and additionally increasing 1% per hour. Overall, the condition has a 90% mortality rate over the course of 12 months.^2,4,5^ Consequently, an early and accurate diagnosis of TAD is essential for its management and few emergency department (ED) presentations are as time-dependent.^6,7^

Unfortunately, two studies have grossly estimated that only one in 10,000 emergency department patients actually has this condition.^6,7^ This makes TAD a condition that every emergency physician will typically see only several times during their careers. Consequently, no physician will see TAD frequently enough to become expert regarding the condition or in making the diagnosis. In fact, TAD has been shown to be initially misdiagnosed in 15-43% of cases.^6,7^ Authors have asserted that even the most experienced expert clinicians remain prone to making a difficult or delayed diagnosis or failing to ever diagnose the condition.^8^ In summary, TAD is an especially uncommon deadly disease with protean symptomatology.

Those ED physicians seeing patients with possible TAD symptoms need to consider the radiation exposure of CT scans, healthcare system costs, cost of incidental findings, and time spent in ED as factors which may preclude the work-up of many patients lacking a realistic possibility of having TAD.^6^ Still, clinicians should conduct an initial work-up for TAD when an ED patient with this prospective condition presents for treatment with these types of symptoms. Clinicians should first use the same initial tools: patient history and a physical examination. It is important that clinicians seek descriptors of the type and nature of the patient’s pain that will help lead them to determine the proper work-up for a timely diagnosis.^7,8^

Historically, medical educators have indicated that patients with TAD typically describe their pain with the adjectives of “tearing” or “ripping.”^9,10^ Tintinalli and colleagues characterized aortic dissection symptoms as a ripping or tearing sensation accompanied by a sense of impending doom.^11^ In 2012, another group stated that TAD patients typically present with abrupt onset of tearing or stabbing chest pain.^12^ The authors’ clinical experiences, however, have challenged the classic belief that these descriptors would be used by many patients with acute TAD. In response, this retrospective pilot project was conducted to examine what proportion of patients with later confirmed TAD had actually used the classic words “ripping” or “tearing” to describe their pain when presenting to an ED.

## METHODS

The authors initiated a systematic retrospective chart review of patients who had presented to the EDs of two Michigan hospitals. The first hospital was a suburban hospital with an annual census of 60,000 patients. The authors reviewed the charts from this facility from years 1997 through 2007. The second hospital was a community urban hospital with an annual census or approximately 27,500 patients. The authors reviewed this system’s charts after the implementation of their electronic health record (EHR) on January 2011 through June 2015.

Initially, the authors electronically searched medical charts by using specific search terms in the discharge diagnosis EHR field such as “aortic dissection,” “thoracic aortic dissection,” or “dissection.” The authors then systematically audited the initial group of identified charts for documentary evidence of TAD. The inclusion criteria for patients to be included in the project sample included confirmation of a TAD by: a) radiographic or angiographic testing, b) cardiac catheterization, c) surgical confirmation, or d) postmortem examination.

Next, the authors inspected all charts with a confirmed TAD. They only examined those areas of the chart where the physician and patient could not have already known patients’ TAD diagnosis. These documentation areas included ambulance transfer notes, notes taken by nurses at initial ED triage, initial bedside ED nursing assessments and the initially treating ED physician notes or dictations. The authors then searched for any notations in these documentation sources in which the patient had described their pain as “ripping” or “tearing.”

## RESULTS

The initial EHR search identified 58 potential patients with TAD discharge diagnoses that indicated they might be included in the chart audit sample. Of this number, the authors excluded 20 (34.5%) patients since they had experienced an aneurysm without rupture or dissection. One (1.7%) patient was excluded with an initial diagnosis of TAD but no thoracic dissection as later found on radiographic studies (i.e., an abdominal aortic dissection). Five (8.6%) more patients were excluded because they were direct hospital admits without ED records. The authors excluded one (1.7%) additional patient because their dissection had occurred after admission to the hospital floor. Finally, two (3.4%) more patients were excluded because they had transferred from other hospitals and their ED records were not readily available.

Ultimately, the medical records of 29 (50.0% of initially pulled) patients met inclusion criteria for evaluation. In summary, the authors’ review of these 29 patient charts failed to show a single documented use of the words “ripping” or “tearing” in patient’s description of their pain in any designated narrative or diagnostic documentation areas.

## DISCUSSION

A patient’s initial history is a key element in making a diagnosis of TAD.^12^ How patients describe their presenting symptoms are key elements in how clinicians’ decide whether to pursue TAD as a possible differential diagnosis. Historically and contemporaneously, physicians have been instructed that patients will most often use the words “ripping” and “tearing” to describe TAD pain.^9,10^ The results of this pilot project calls that diagnostic principle into question since we found no documented use of either of these symptomatic descriptors among the charts of sample patients examined.

Unlike the larger IRAD study,^3^ our smaller-scale chart audit project results demonstrate no use of the terms “ripping” and “tearing.” This may reflect the methodology we used since we only audited the documentation sections that clinicians had completed before a TAD diagnosis had been confirmed. One could easily imagine a situation in which clinicians who already knew patients’ TAD diagnoses may have asked them whether their pain felt like “ripping or tearing” to which the patient could have simply agreed.

## CONCLUSION

It will remain imperative for emergency medicine and other physicians to make time-dependent diagnoses of TAD in a timely fashion since the mortality risks are so very high. Judging from these findings, practitioners should not exclusively listen for the trigger words “ripping” or “tearing” when potential TAD patients are describing their symptoms. Judging from the results of this and prior studies providers may be more clinically shrewd to conduct a more comprehensive evaluation of other subjective pain descriptors.

Medical educators have traditionally taught students that the terms “ripping” and “tearing” are the typical descriptors patients will offer when describing the pain of TAD. Our project findings support our initial clinical impression that patients would not routinely use these terms. It can be especially problematic when physicians rely on these specific descriptors, leading unwary physicians toward a delayed or even missed TAD diagnosis.

### Conflict of Interest

The authors declare no conflict of interest.
